# The Psychometric Properties of the Post‐Traumatic Stress Disorder Questionnaire (PCL‐5) for Cancer Patients in Iran

**DOI:** 10.1002/cnr2.70331

**Published:** 2025-09-20

**Authors:** Maryam Hasannezhad Reskati, Hamid Sharif‐Nia, Seyed Hamzeh Hosseini, Hossein Azadeh, Behnoush Yazdirad, Reza Alizadeh‐Navaei, Forouzan Elyasi

**Affiliations:** ^1^ Phd Educational Psychology, Research Ethics Committee, Imam Khomeini Hospital Mazandaran University of Medical Sciences Sari Iran; ^2^ School of Nursing and Midwifery Amol Mazandaran University of Medical Sciences Sari Iran; ^3^ Psychosomatic Research Center Mazandaran University of Medical Sciences Sari Iran; ^4^ Department of Psychiatry, Faculty of Medicine Mazandaran University of Medical Sciences Sari Iran; ^5^ Department of Internal Medicine, Rheumatology Division, Orthopedic Research Center Mazandaran University of Medical Sciences Sari Iran; ^6^ Gastrointestinal Cancer Research Center, Non‐Communicable Diseases Institute Mazandaran University of Medical Sciences Sari Iran; ^7^ Gastrointestinal Cancer Research Center Mazandaran University of Medical Sciences Sari Iran; ^8^ Sexual and Reproductive Health Research Center, Psychiatry and Behavioral Sciences Research Center, Addiction Institute Mazandaran University of Medical Sciences Sari Iran; ^9^ Faculty of Medicine Mazandaran University of Medical Sciences Sari Iran

**Keywords:** cancer, nurses, post‐traumatic stress, psychometric

## Abstract

**Background:**

Cancer is known as a common chronic disease that many aspects of its diagnosis and treatment may manifest traumatic episodes and lead to the post‐traumatic stress disorder (PTSD) associated with cancer. Avoidance symptoms in PTSD often delay individuals from seeking professional assistance. Therefore, the PTSD Checklist for the Diagnostic and Statistical Manual of Mental Disorders‐5th Edition or DSM‐5 (PCL‐5) can evaluate PTSD conceptually and psychometrically instead of avoidance responses.

**Aims:**

Accordingly, this research aimed to explore the psychometric features of the PCL‐5 Persian version in the population of gastrointestinal cancer patients in Iran.

**Methods:**

As the instrument of the research, the Persian version of PCL‐5 was conducted on 486 gastrointestinal cancer patients who referred to Baghban Clinic, the cancer research center of “REDACTED” University of Medical Sciences, and Imam Khomeini Hospital in Sari from July 2021 to May 2022. The used questionnaire survey included two sections. The first part consisted of questions regarding participants' profiles, such as age, gender, and job status. In the second section, the Posttraumatic Stress Disorder item 20 was used to measure (PCL‐5) Participants were asked to respond to each statement with a 5‐point range Likert scale from 0 to 4.

**Results:**

Exploratory factor analysis showed four‐factor structures for PCL‐5, explaining 43.17% of the total variance. According to the mentioned analysis, each fit index verified the model fit. All internal consistency coefficients were also predicted to be acceptable reliability. It can be concluded from the data that highlighted good psychometric features for PCL‐5, suggesting the effectiveness of this scale for the screening of PTSD in gastrointestinal cancer patients.

**Conclusion:**

This study indicates the effectiveness and usefulness of the Persian version of the PCL‐5 scale for assessing post‐traumatic stress in the cases suffering from gastrointestinal cancer.

## 1 Introduction

Cancer is a life‐threatening condition [1]. Despite significant advances in therapeutic measures, patients may experience episodes of psychological distress [2], thus leading to trauma and emergence of avoidance behaviors, intrusive thoughts, a negative change in mood and cognition, and finally arousal elevation [3, 4]. Such symptoms can highlight the presence of post‐traumatic stress disorder (PTSD) [5].

One of the popular misconceptions is that PTSD merely occurs in acute situations, physical trauma, or war conditions [6]. According to the diagnostic criteria for PTSD in the latest version of the PTSD Checklist for DSM‐5 (PCL‐5), every life‐threatening disease or weakening medical condition is not certainly categorized among the traumatic events, and medical events such as cancer qualify as traumatic ones, which will be experienced suddenly and catastrophically [3]. However, a variety of dimensions of the diagnosis and treatment of cancers may manifest traumatic episodes and lead to PTSD associated with cancer, such as diagnostic tests, the stressful waiting period, time of hearing or seeing bad news, as well as worrisome treatments [7]. Cancer is so frightening for some people that they are afraid to even say the word cancer [8]. Acute stress disorder symptoms have been viewed as the pathological conditions in the case of persistence for more than a month after the trauma, with side effects like functional impairment and distress [9], and are often confused with anxiety or depression [6].

The prevalence of PTSD in patients with different cancers ranges from 3% in breast cancer [10], 11.8% in head and neck cancer [11] to 36%–45% in ovarian cancer [12]. Studies with similar groups also reported variable PTSD prevalence [10, 13]. In fact, reliable and valid equipment must be employed to diagnose PTSD exactly, and one reason for the varying prevalence reports appears to be instrument‐related. Some studies have employed the Structured Clinical Interview for DSM‐5 Disorders (SCID‐5) [14]. The SCID‐5 has been presented as one of the clinician‐administered structured clinical interviews. It is time‐consuming and cannot be implemented in cases with no knowledgeable mental health experts. Other studies used an affordable screening tool (i.e., the PTSD Checklist‐Civilian Version [PCL‐C]) [15, 16]. Multiple publications utilized The Impact of Event Scale (IES), which is one of the self‐reports of intrusive thoughts [17]; hence, it could be useful to assess the PTS symptoms; however, it has not been developed for PTSD assessment.

According to the literature, chronic and also disastrous psychological and inter‐personal consequences of the PTSD require efficient and laborious treatments [18, 19]. Avoidance symptoms in the PTSD often hamper or prevent finding and receiving specialized supports. Although no certain treatment has been developed for PTSD in different kinds of cancers, therapeutic methods applied for PTSD patients may be useful for alleviating distress in cases with cancer as well as its survivors [20, 21].

Some of the tools available to diagnose PTSD include the IES‐R [22, 23], Mississippi PTSD Scale (MCCP) [24], Post Trauma Symptoms Scale (PTSS) [25], and PCL‐C [26]. Moreover, PTSD in PCL‐5 was changed from the category of the anxiety disorders to one of the new classes named “Trauma and Stressor‐related Disorders.” In addition, Criterion A2 (response involving helplessness, posttraumatic fear, and horror) has been deleted and new symptoms (i.e., continual and distorted self‐blaming or blaming others, continual negative emotions, and or self‐destructive or inattentive behaviors) were introduced, and numerous other items have been modified [3], and PCL‐5 version was designed accordingly [27].

PCL‐5 has been investigated in different countries such as Italy [28], Iraq [29], Brazil [30] as well as Canada [31]. It was also validated in Zimbabwe on HIV patients [32] and by Varmaghani in Iran on citizens of Tehran who had experienced excessive stress [33].

Some scholars showed the concentration of religion on a person's beliefs, coping, and behaviors [34]. In many studies, Muslim cancer patients declared that God had powers to control their circumstances and lives. They also accepted the fact that they are not able to change their fate. Such beliefs and ideas can assist Muslims in overcoming cancer‐related negative experiences and emotions. The cases admitted the importance of their absolute beliefs in the forgiveness and compassion of God as the spiritual resources and religious actions [35, 36, 37]; on the other hand, there may be religious obstacles for many Muslims. In fact, their possible feeling is that God has predestined all events in life, and even different kinds of cancers, or cancer is the hurt to compensate for sins committed in the past [38]. Coping with stress in different cultural groups can depend on culture. On the other hand, religion in different societies like Iran plays a significant role in adopting a person's coping style in response to problems. In addition, there is a newer version of the PCL and that no studies have been conducted on cancer patients, who constitute a large group of patients, in this field as far as we know. Accordingly, the current attempt sought to evaluate the factor structure and validity and reliability of the Persian version of PCL‐5 among the population of gastrointestinal cancer patients in Iran.

## 2 Methods

### 2.1 Research Design

The present methodological research adopted a cross‐sectional design to evaluate the psychometric features of PCL‐5 amongst Iranians with gastrointestinal cancer. The inclusion criteria included patients over 18 years of age, literacy, confirmation of gastrointestinal cancer by the treating physician, and exclusion criteria: physical inability to respond, accompanying another type of physical illness such as other cancers, multiple sclerosis (MS), spinal cord and brain tumors, or chronic mental disorders like schizophrenia, bipolar disorder, and severe emotional disorders. The questionnaire survey was conducted in the cases who had been referred to Baghban Specialized Oncology Clinic as well as Imam Khomeini Hospital in Sari city, northern Iran, from July 2021 to May 2022. In total, 486 were included in the present research.

### 2.2 Measures

The used questionnaire survey included two sections. The first part consisted of questions regarding participants' profiles, such as age, gender, and job status. In the second section, the Posttraumatic Stress Disorder item 20 was used to measure (PCL‐5) Participants were asked to respond to each statement with a 5‐point Likert scale from 0 (not true at all) to 4 (true nearly always).

#### 2.2.1 PCL‐5 Questionnaire

As mentioned earlier, the translated version of the PCL‐5 was a self‐reported 20‐item scale, which addressed the evaluation of the severity, incidence, as well as 20 symptoms of PTSD. The DSM‐5 (PCL‐5) also includes four subscales matching the four signs of DSM‐5 disruption, and items were ranked on the Likert scale [27].

### 2.3 Procedure

Considering ethical issues, a written permit to apply the PCL‐5 was received from the scale's developer, Dr. Frank Weathers, using an email communication. In the next step, a forward‐backward translation approach was implemented, and two English–Persian translators were invited to translate the PCL‐5 to Persian. The two translators translated the PCL‐5 into a Persian version independently. Afterwards, these two Persian versions of the PCL‐5 were reviewed and commented on by some experts, such as a number of authors of the present research and another two professional translators, to form a single Persian version of the PCL‐5. At the end, a single Persian version of the PCL‐5 was also back‐translated by a Persian–English translator to English and confirmed by the experts on the translation accuracy.

### 2.4 Construct Validity and Reliability

The present research utilized both exploratory factor analysis (EFA) and confirmatory factor analysis (CFA) for confirming reliability, construct validity, and factorial structures of the Persian version of PCL‐5. For the purpose of data analyses, the dataset (*n* = 486) was divided into two groups randomly; the first (*n* = 243) was used to conduct EFA with the help of SPSS 27, and the second (*n* = 243) was applied to conduct CFA with the help of AMOS 27.

This research also utilized the Kaiser–Meyer–Olkin (KMO) > 0.8 and robust maximum likelihood (MLR) EFA with Promax rotation. Moreover, Bartlett's test of sphericity was significant (*p* < 0.05) and has been applied for assessing the data utility for running the factor analysis. The analysis determined the factor structure by calculating eigenvalues, which represent the variance in each item accounted for by the factor. The percentage of total variance explained by each factor was calculated by dividing the eigenvalue by the total number of items [39].

The factorial structure of the Persian version of PCL‐5 was following the criteria presented here: communalities > 0, as well as, eigenvalues > 1. Additionally, factor loadings for all of the items in the extracted factors must be > 0.3 [40]. Next, the maximum likelihood CFA was run for validating the factorial structure extracted from the EFA. In the next step, the evaluation of the model fit was performed by a number of fit indices like Chi‐square (*χ* ^2^), goodness‐of‐fit index (GFI) > 0.9, *χ* ^2^/degree of freedom (df) ratio < 4, normed fit index > 0.9, comparative fit index (CFI) > 0.9, incremental fit index (IFI) > 0.9, Tucker–Lewis index (TLI) > 0.9, relative Fit Index > 0.9, standardized root mean square residual < 0.09, and finally the root mean square error of approximation (RMSEA) < 0.08 [41]. Then, we assessed the PCL‐5 Persian version through its discriminant and convergent validities. To meet convergent validity, composite reliability (CR) must be > 0.7, and Average Variance Extracted (AVE) must be > 0.5. Also, for divergent validity, Maximum Shared Variance (MSV) should be less than AVE [42].

### 2.5 Model Comparison Analysis

To evaluate the improvement in model fit from the first‐order to the second‐order CFA, we conducted a DIFFTEST (Δ*χ* ^2^) and calculated the difference in the CFI (ΔCFI). These tests were performed to assess whether the second‐order model provided a statistically significant and meaningful improvement over the first‐order model. Following established criteria [43, 44, 45], a non‐significant Δ*χ* ^2^ (*p* > 0.050) and a ΔCFI < 0.010 were used as cutoffs to support the superiority of the second‐order model.

This research addressed the assessment of construct reliability in relation to its internal consistency (comprise of McDonald's omega and Cronbach's alpha [*α*]), maximum reliability (MaxR), as well as CR. Hence, to achieve excellent construct reliability, we must obtain values > 0.7 for MaxR, Cronbach's alpha (*α*), McDonald's omega, and CR [42].

### 2.6 Multi‐Variate Normality and Outliers

We assessed multi‐ and univariate normality of the data and tested univariate distribution in terms of skewness, kurtosis, as well as outliers. Then, Mardia's coefficient of multivariate kurtosis was employed for assessing the multivariate normality. It should be noted that Mardia's coefficient greater than 8 may be regarded as a sign of divergence from multivariate normality. Consequently, Mahalanobis distance was utilized for detecting the multivariate outliers (*p* < 0.001) [46].

## 3 Results

### 3.1 Characteristics of the Participants

In total, 486 Iranians with gastrointestinal cancer were included in the present research. They included 206 males and 280 females, with the mean age of 49.867 years (SD = 12.506) (Table 1).

**TABLE 1:** Demographic characteristics information.

Variable	EFA		CFA		MD	SD
Age	Minimum	Maximum	Minimum	Maximum	49.72	12.52
	19.00%	76.00%	21.00%	78.00%		

		EFA	Percentage	Total EFA	CFA	Percentage	Total CFA	Total
Gender	Male	110	45.3%	243	95	39.1%	243	486
	Female	133	54.7%		148	60.9%		
Marital status	Single	34	14%	243	31	12.8%	243	486
	Married	209	86%		212	87.2%		
Education level	High school diploma and below	164	67.5%	243	187	77.0%	243	486
	Associate's degree	33	13.6%		13	5.3%		
	Bachelor's degree (BA)	39	16.0%		34	14.0%		
	Master's degree (MA) and higher	7	2.9%		9	3.7%		
Occupation	Homemaker	98	40.3%	243	121	49.8%	243	486
	Public servant	33	13.6%		22	9.8%		
	Factory worker	8	3.3%		3	1.2%		
	Pensioner	50	20.6%		43	17.7%		
	Self‐employed	54	22.2%		54	22.2%		
Treatment stage	Chemotherapy	146	60.1%	243	159	65.4%	243	486
	Radiotherapy	13	5.3%		7	2.9%		
	Pharmacotherapy	59	24.3%		58	23.9%		
	Follow‐up	10	4.1%		14	5.8%		
	Chemotherapy plus radiotherapy	8	3.3%		5	2.1%		
	Surgery	7	2.9%		0	0.0%		
Type of cancer	Stomach	91	37.4%	243	86	35.4%	243	486
	Intestinal (namely, ileum, duodenum, duodenum, and jejunum)	33	13.6%		29	11.9%		
	Colon (viz. rectum and sigmoid)	81	33.3%		65	26.7%		
	Esophageal	6	2.5%		28	11.5%		
	Liver and gallbladder	23	9.5%		19	7.8%		
	Pancreatic	9	3.7%		14	5.8%		
	Mouth	0	0.0%		1	0.4%		
Duration of disease	Below 6months	102	42.0%	243	86	35.4%	243	486
	6–12 months	40	16.5%		49	20.2%		
	1–5 years	71	29.2%		88	36.2%		
	5–10 years	28	11.5%		15	6.2%		
	10–20 years	0	0.0%		2	0.8%		
	Above 20 years	2	0.8%		3	1.2%		
History of other physical illnesses	Yes	111	45.7%	243	111	45.7%	243	486
	No	132	54.3%		132	54.3%		
History of mental problems	Yes	17	7.0%	243	29	11.9%	243	486
	No	226	93.0%		214	88.1%		
Family history of cancer	Yes	106	43.6%	243	92	37.8%	243	486
	No	137	56.4%		151	62.1%		
Place of residence	City	163	67.1%	243	155	63.8%	243	486
	Village	80	32.9%		88	36.2%		

### 3.2 Reliability and Validity

EFA results with the Promax rotation (*n* = 243) on the Persian version of PCL‐5 are presented in Table 2. The results showed that the KMO equals 0.937, and the Bartlett's Test of Sphericity is significant (*p* < 0.001, 5012.88, df = 190), revealing the data utility for doing the factor analysis. Finally, we could extract four factors that consisted of 20 items and justified 43.17% of the total variance.

**TABLE 2:** The results obtained for EFA & internal consistency on the 4 factors Persian version post‐traumatic stress disorder (*n* = 243).

Factor	Items	Factor loading	*h* ^2^	*λ*	% Variance	Internal consistency
Re‐experiencing	Q_2_. Repeated, disturbing dreams of the stressful experience?	0.962	0.827	2.818	14.10	*α* = 0.872 Ω = 0.880 AIC = 0.588
	Q_1_. Repeated, disturbing, and unwanted memories of the stressful experience?	0.940	0.718			
	Q_4_. Feeling very upset when something reminded You of the stressful experience?	0.643	0.626			
	Q_3_. Suddenly feeling or acting as if the stressful experience were actually happening again (as if you were actually back there reliving it)?	0.632	0.392			
	Q_5_. Having strong physical reactions when something reminded you of the stressful experience (e.g., heart pounding, trouble breathing, sweating)?	0.443	0.536			
Negative alterations in cognition and mood	Q_9_. Having strong negative beliefs about yourself, other people, or the work (e.g., having thoughts such as: I am bad, there is something seriously wrong with me, no one can be trusted, the world is completely dangerous)?	0.817	0.595	2.464	12.32	*α* = 0.849 Ω = 0.850 AIC = 0.485
	Q_10_. Blaming yourself or someone else for the stressful experience or what happened after it?	0.779	0.511			
	Q_11_. Having strong negative feelings such as fear, horror, anger, guilt, or shame?	0.632	0.519			
	Q_15_. Irritable behavior, angry outbursts, or acting aggressively?	0.565	0.577			
	Q_14_. Trouble experiencing positive feelings (e.g., being unable to feel happiness or have loving feelings for people close to you)?	0.559	0.377			
	Q_13_. Feeling distant or cut off from other people?	0.399	0.463			
Hyper‐arousal	Q_18_. Feeling jumpy or easily startled?	0.886	0.723	1.958	9.80	*α* = 0.820 Ω = 0.824 AIC = 0.435
	Q_17_. Being “superalert” or watchful or on guard?	0.678	0.389			
	Q_19_. Having difficulty concentrating?	0.520	0.490			
	Q_20_. Trouble falling or staying asleep?	0.447	0.404			
	Q_16_. Taking too many risks or doing things that could cause you harm?	0.386	0.393			
	Q_12_. Loss of interest in activities that you used to enjoy?	0.307	0.409			
Avoidance	Q_7_. Avoiding external reminders of the stressful experience (e.g., people, places, conversations, activities, objects, or situations)?	0.974	0.874	1.388	6.95	*α* = 0.769 Ω = 0.779 AIC = 0.525
	Q_6_. Avoiding memories, thoughts, or feelings related to the stressful experience?	0.581	0.455			
	Q_8_. Trouble remembering important parts of the stressful experience?	0.320	0.505			

Abbreviations: *h* ^2^, communalities; *λ*, eigenvalues.

**FIGURE 1:**
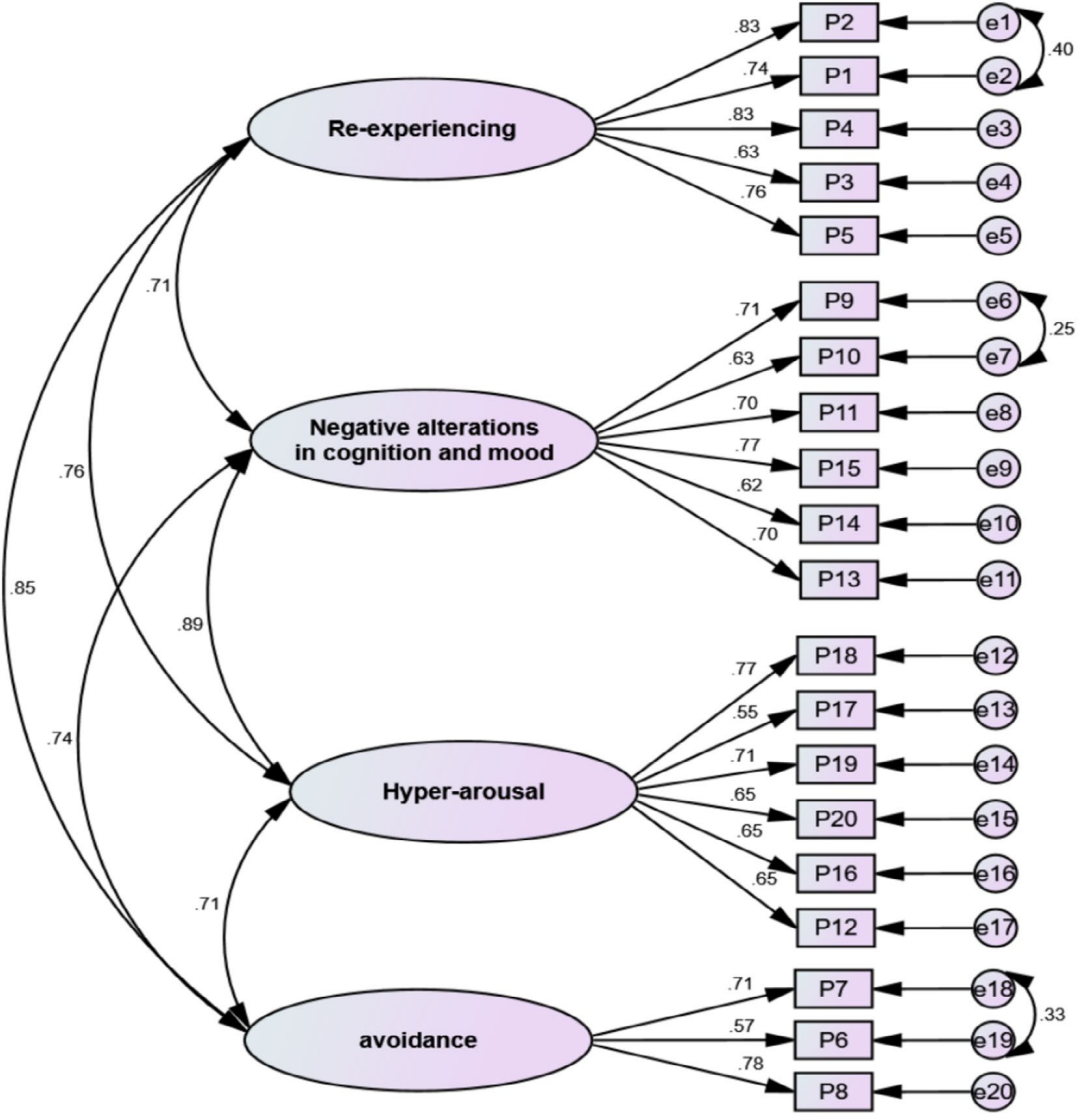
The first‐order CFA of the PTSD.

Next, maximum likelihood CFA (*n* = 243) conducted for validating the factorial structures determined by EFA (Table 3). With regard to Figure 1, to improve this model, three pairs of measurement errors were allowed to freely co‐vary (e1 to e2, e6 to e7, and e18 to e19). Moreover, factor loadings for each item ranged between 0.55 and 0.83 and were significant. The final four‐factor model was also shown to fit the data completely after reviewing the modification indicators (${\chi^{2}}_{\left(161\right)}$ = 482.318, *p* < 0.001, *χ* ^2^/df = 2.996, GFI = 0.910, CFI = 0.934, IFI = 0.935, TLI = 0.923, RMSEA (90% CI) = 0.064 [0.058, 0.071]).

**TABLE 3:** The indicators of discriminant and the convergent validities in the first and second‐order CFA model: (*n* = 243).

The first‐order CFA				
	CR	AVE	MSV	MaxR (*H*)
Re‐experiencing	0.871	0.577	0.715	0.884
Negative alterations in cognition and mood	0.845	0.478	0.795	0.851
Hyper‐arousal	0.825	0.442	0.795	0.835
Avoidance	0.731	0.479	0.715	0.754
The second‐order CFA				
PTSD	0.939	0.794	—	0.944

Coefficients of McDonald's omega, Cronbach's alpha (*α*), MaxR, as well as CR for each factor were determined to be > 0.7, illustrating the satisfied internal consistency and construct reliability. Moreover, the AVE for four factors was lower than the true threshold of 0.5 or MSV, and AVE was considered one of the strict measurements for convergent validity. Furthermore, CR > 0.7 could be applied to assess convergent validity in the psychological investigations. Therefore, convergent validity has been achieved in this study as CR for the two factors was > 0.7 (Figure 1).

**FIGURE 2:**
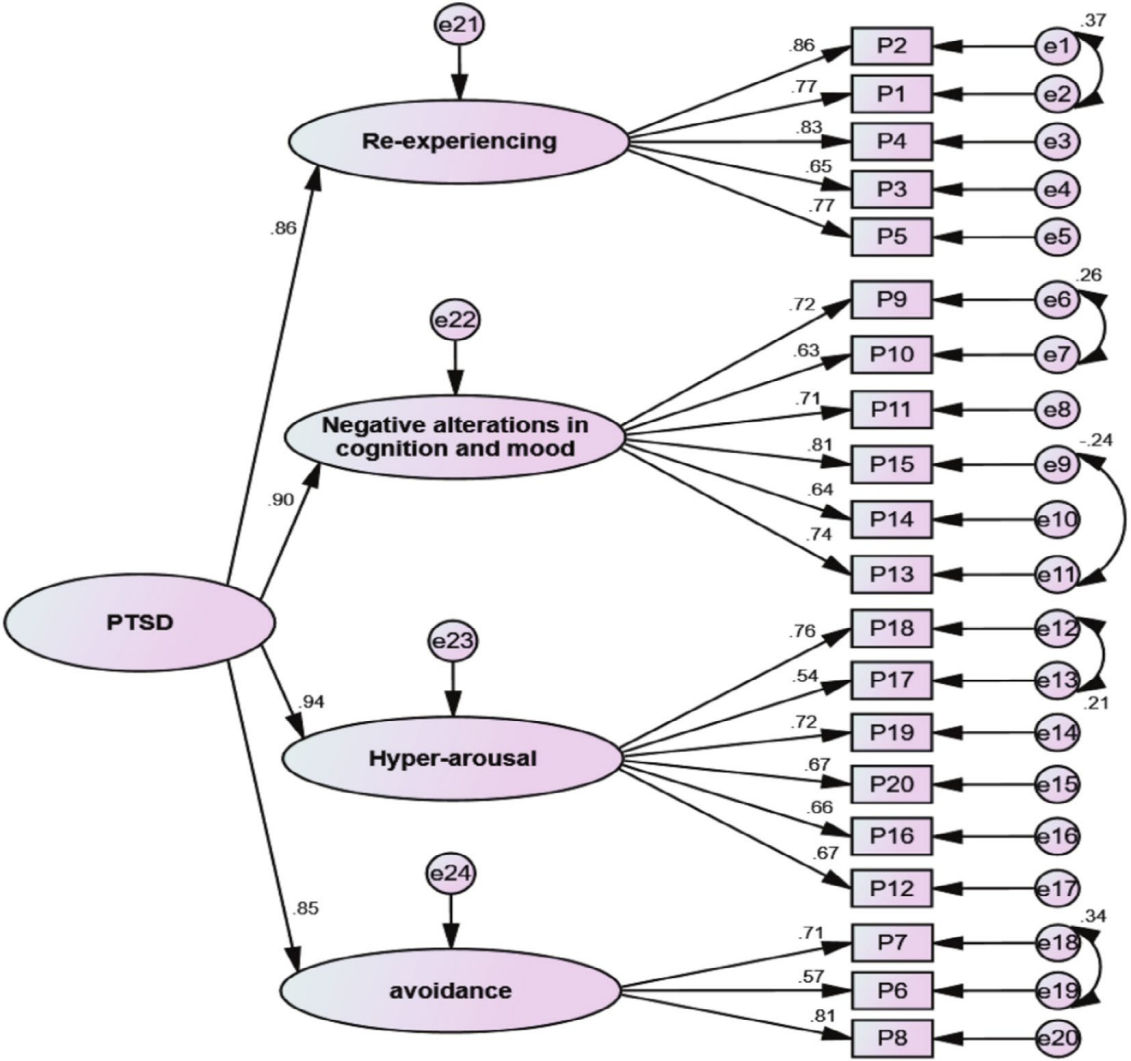
The second‐order CFA of the PTSD.

Given that MSV for all of the factors was greater than AVE, the second‐order CFA was run. The second‐order CFA model was shown to fit the data completely after reviewing the modification indicators (${\chi^{2}}_{\left(162\right)}$ = 516.365, *p* < 0.001, *χ* ^2^/df = 3.187, GFI = 0.911, CFI = 0.928, IFI = 0.928, TLI = 0.915, RMSEA (90% CI) = 0.067 [0.064, 0.074]) (Figure 2).

### 3.3 Model Comparison

To evaluate the improvement in model fit from the first‐order to the second‐order CFA, we conducted a DIFFTEST (Δ*χ* ^2^) and calculated ΔCFI. The second‐order model demonstrated a significant improvement in fit (Δ*χ* ^2^ = 34.047, *p* < 0.001; ΔCFI = 0.006), meeting the criteria for model superiority (ΔCFI < 0.010). This supports the hierarchical structure of the four‐factor model as a better representation of the data.

## 4 Discussion

Our study revealed that the Persian version of PCL‐5 has 20 items in four subscales (re‐experience, negative change in cognition and moods, hyper‐arousal, avoidance) and these four factors explain about half of the total variances of PTSD out of gastrointestinal cancer patients.

Moreover, the high value obtained for Cronbach's alpha (< 0.82), the mean correlation of the items, as well as McDonald's omega (< 0.83) revealed acceptable internal consistency for the four factors of the scale. Internal consistency also showed the same outputs as the original scale. Calculating McDonald's omega is the advantage of this study because it is independent of the size of samples and the number of items.

In addition, outputs from Max‐R and CR (> 0.81) confirmed good reliability for the Persian version of PCL‐5. Factor loading in CFA estimates CR [47]. The EFA findings identified four factors, the first of which was a five‐item re‐experiencing.

As opposed to the single traumatic episodes, which cause PTSD, different kinds of cancers usually behave as a continuing and persistent stressor [48]. Symptoms of re‐experience, such as involuntary memories, distressing dreams, dissociative reactions, flashbacks, or intensely distressing psychological or physiological responses to reminders of the trauma [3], are likely to be repeated with each follow‐up of treatment procedures or periodic tests.

The second factor is negative alterations in cognition and mood, with six items. Due to the difficult conditions of the disease, treatment, and side effects, patients are faced with many ambiguities probably inducing negative mood states [49].

Iran is a country where the majority of its people are religious, and religion is expected to play a moderating role in these ambiguities and reduce negative emotions, although studies confirm the positive effect of religious and spiritual practices on cancer patients [50].

Several religious ideas and beliefs probably direct sufferers to the interpretation of their illnesses as acceptance, punishment, and or refusal from a divine resource like God [51]. Therefore, negative religious coping probably causes the experiences of emotions of neglect, guilt, and emotional suffering/distress in people and lessens their ability to cope with difficult life episodes [52].

The third factor, hyper‐arousal, has also six items.

Pain and suffering caused by the disease, worry about the future of the family members, fear of death, complications caused by the treatment of the disease, etc., are all effective in reducing the mental health of people with cancer [53] and can cause emotional arousal in the person. The use of strategies that enable people to maintain the ability to adapt to the disease process [54] can improve the mental health of the affected person.

The fourth factor is avoidance. Experiential avoidance represents the excessive negative assessment of undesired personal emotions, thoughts, sensations, and disclinations for experiencing such private events and controlling or escaping them [55]. It often leads to behavioral avoidance or engaging in behaviors that interfere with the individual's performance. In addition to effects such as substance abuse, overeating, or self‐harm, experiential avoidance can have subtle consequences on a person's relationships in everyday life and hinder the pursuit of a career path, goals, values, and standards that are important to a person [56]. Apparently, because any kind of cancer is usually considered as one of the chronic and persistent stressors, the avoidance coping strategy is less used due to the nature of the disease and its treatment. In addition, cancer patients are constantly applying spiritual and religious coping skills in facing their illness [57]. Intrusive memories appear spontaneously in PTSD patients [58]. Religious beliefs, which are controlled processes, probably affect the intrusive memories, which seem to be a spontaneous process, and thus cause this factor to shift to the fourth factor.

The number of item and factor of the Persian version of PCL‐5 was as the same as the original one. The differences between the Persian and original versions were the second factor or avoidance, which became the fourth factor. Additionally, the question “Do you have trouble in recalling significant parts of the stressful experiences?” moved from the factor of negative alterations in cognition and mood to the factor of avoidance.

Even though experts in the field have proposed the availability of the respondents as a main benefit of convenience sampling, a certain population can be oversampled or some people in the target population may not participate in the respective sample. In this research, considering that we studied gastrointestinal cancer patients who have been referred to Baghban Clinic, the cancer research center of Mazandaran University of Medical Sciences, and Imam Khomeini Hospital in Sari, one of the northern cities of Iran (both of which are government centers), the patients of private centers could not access the data collection form. Another limitation of our study was the validation of this questionnaire only in one group of cancer patients, so these results may be different in patients with other cancers. This research is suggested to be implemented in other types of cancer as well. About the generalizability of the results of this research, it should be noted that because the participants were selected from a specific geographical area or subgroup of patients with gastrointestinal cancer, the results might not be generalizable to all populations. Veterans and other people may have experienced different types of trauma, which could influence their responses to the questionnaire. The research might have limitations in accounting for this diversity. Response bias is another limitation of the research. Responses from gastrointestinal cancer patients might be influenced by social desirability or psychological biases, which could have been insufficiently addressed. Another important limitation is that for assessing the convergent and divergent validity only the questionnaire was used, and the psychiatric clinical interview according to the DSM‐5‐TR was not used.

## 5 Conclusions

As mentioned earlier, the research results indicate the effectiveness and usefulness of the Persian version of the PCL‐5 scale for assessing post‐traumatic stress in cases suffering from gastrointestinal cancer. This scale helps healthcare providers, nurses, psychiatrists, as well as psychologists identify and screen high‐risk cases and adopt preventive interventions to minimize the development of irreversible PTSD complications.

## Author Contributions

Maryam Hasannezhad Reskati designed the study and did the literature search, data gathering, interpreted the findings, drafting the manuscript, and editing the manuscript. Reza Alizadeh‐Navaei, Behnoush Yazdirad and Hossein Azadeh contributed in data gathering. Hamid Sharif‐Nia analyzed the data, interpreted the findings and editing the manuscript. Seyed Hamzeh Hosseini contributed in editing the manuscript. Forouzan Elyasi participated in study design, collecting the data, interpreted the findings and editing the manuscript. All authors read and approved the final manuscript.

## Ethics Statement

Ethics Committee affiliated with Mazandaran University of Medical Sciences approved this report [IR. MAZUMS.IMAMHOSPITAL.REC 1400.078].

## Consent

Informed consent was obtained from all participants included in the study.

## Conflicts of Interest

The authors declare no conflicts of interest.

## Data Availability

The data that support the findings of this study are available on request from the corresponding author. The data are not publicly available due to privacy or ethical restrictions.
